# Electrospun Nanofiber-Based Membranes for Water Treatment

**DOI:** 10.3390/polym14102004

**Published:** 2022-05-13

**Authors:** Yixuan Tang, Zhengwei Cai, Xiaoxia Sun, Chuanmei Chong, Xinfei Yan, Mingdi Li, Jia Xu

**Affiliations:** Key Laboratory of Marine Chemistry Theory and Technology, Ministry of Education, College of Chemistry and Chemical Engineering, Ocean University of China, Qingdao 266100, China; yixuantang06@163.com (Y.T.); caizhengwei2021@163.com (Z.C.); sunxxia0812@163.com (X.S.); 18264722913@163.com (C.C.); yanxinfei99@163.com (X.Y.); mingdili08@163.com (M.L.)

**Keywords:** electrospinning, electrospun nanofiber-based membranes (ENMs), nanofiber layer, membrane fabrication, water treatment

## Abstract

Water purification and water desalination via membrane technology are generally deemed as reliable supplementaries for abundant potable water. Electrospun nanofiber-based membranes (ENMs), benefitting from characteristics such as a higher specific surface area, higher porosity, lower thickness, and possession of attracted broad attention, has allowed it to evolve into a promising candidate rapidly. Here, great attention is placed on the current status of ENMs with two categories according to the roles of electrospun nanofiber layers: (i) nanofiber layer serving as a selective layer, (ii) nanofiber layer serving as supporting substrate. For the nanofiber layer’s role as a selective layer, this work presents the structures and properties of conventional ENMs and mixed matrix ENMs. Fabricating parameters and adjusting approaches such as polymer and cosolvent, inorganic and organic incorporation and surface modification are demonstrated in detail. It is crucial to have a matched selective layer for nanofiber layers acting as a supporting layer. The various selective layers fabricated on the nanofiber layer are put forward in this paper. The fabrication approaches include inorganic deposition, polymer coating, and interfacial polymerization. Lastly, future perspectives and the main challenges in the field concerning the use of ENMs for water treatment are discussed. It is expected that the progress of ENMs will promote the prosperity and utilization of various industries such as water treatment, environmental protection, healthcare, and energy storage.

## 1. Introduction

Clean water is known as one of the essential resources for human utilization. According to a United Nations report, by 2050 nearly six billion people worldwide will suffer from clean water scarcity [1]. Numerous plants, among other things, such as municipal/industrial wastewater purification and seawater desalination, were established to ensure a sufficient clean water supply. Currently, water purification plants predominantly involve physical approaches (filtration, sedimentation, and centrifugation), chemical approaches (flocculation, coagulation, and oxidation), biological approaches (anaerobic and aerobic digestions), and reverse osmosis and flash distillation technologies, aiming at the reduction and removal of contaminants for clean potable water [2,3]. Among these techniques, membrane technology, with unique advantages of non-phase change, low energy consumption, high-quality water supply, space-saving, and easy integration with other processes, has aroused dramatic interest in academia and industry. Membrane separation processes can be classified according to separation principles and membrane properties. Based on the pressure-driven separation process, membranes can be classified as microfiltration, ultrafiltration, nanofiltration, reverse osmosis, forward osmosis, and pressurized delayed infiltration. According to the thermal-driven separation process, membranes can be divided into pervaporation and membrane distillation.

The rapid expansion of nanotechnology, such as nanomaterials with extraordinary physical and chemical properties, could assist in satisfying the demand for high-quality purification applications. The nanomaterials include zero-dimensional nanoparticles (all three dimensions are in the range of 1–100 nm, for example, quantum dots), one-dimensional materials (one of the dimensions is on a nanometer scale, examples include nanofibers, nanorods, nanotubes, nanowires), and two-dimensional nanosheets (all three dimensions are >100 nm) [4]. Among these, nanofibers, especially synthesized by electrospinning for a predominant electroactive phase, contribute extraordinary features to nanotechnology’s development [5]. Nanofibers are unique among the numerous types of nanomaterials due to their remarkably high specific surface area and porosity. In general, there are many techniques to produce nanofibers, such as bicomponent extrusion, electrospinning, melt blowing, phase separation, centrifugal spinning, drawing, self-assembly, and template synthesis (Table 1). The electrospinning (ES) technique is a cost-effective, simple and economic strategy for nanofiber production with the most control over parameters of nanofibers varying in size, shape, and doping [6]. Relatively symmetrical and homogeneous-structured nanofiber scaffolds can be developed alongside the production of membranes with excellent performance in water purification operations [7]. Membranes mainly fabricated via a sole or integrated ES technique, typically known as electrospun nanofiber-based membranes (ENMs), consist of nanofiber layers with overlapped nanofibers of diameters ranging from several nanometers to a few microns. ENMs have been regarded as one of the most promising orientations for energy storage, health care, electricity generation, biotechnology, and environmental applications, benefiting from these features, especially water purification and desalination [8].

**Figure 1:**
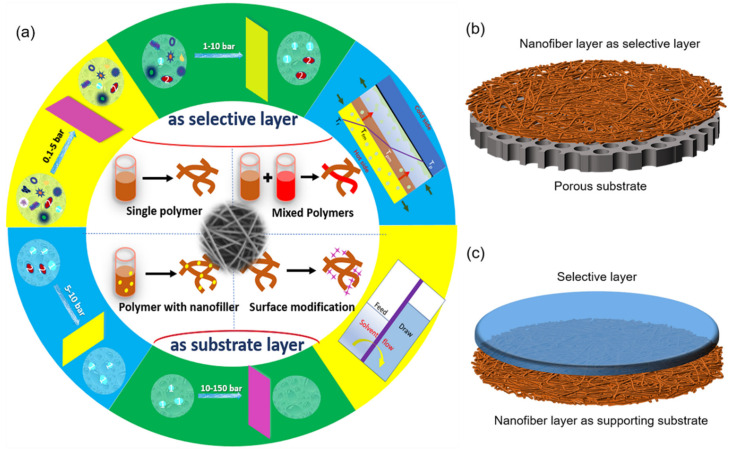
(**a**) Preparation and application of ENMs; (**b**,**c**) ENMs schematic images of two roles of nanofiber layers: served as selective layer (**b**) and supporting substrate (**c**).

The efficiency of a membrane-based desalination system, particularly its permeability (water flux) and separation efficiency (solute rejection), depend considerably on the membrane properties and performance [9]. For increasingly enhanced separation requirements to be met, the ENMs require constant improvements and optimization for increased efficiency in both pressure-driven and thermal-driven processes with less consumption. According to our best knowledge, few studies have focused on the production and application of ENMs, with an eye to functionalized nanofibers for specific applications. As shown in Figure 1a, ENMs can be divided into the following two categories according to the roles of electrospun nanofiber layers: (i) nanofiber layer serving as a selective layer, (ii) nanofiber layer serving as supporting substrate. The synthesis method of ENMs can be classified as single polymers, mixed polymers, polymers with nanofiber, and surface modifications. ENMs mainly have the following application fields: microfiltration, ultrafiltration, nanofiltration, reverse osmosis, forward osmosis, pervaporation, membrane distillation, etc. Regarding applications in water purification and desalination, the position of the nanofiber layer in ENMs have suggested that there are two types of ENMs, with the nanofiber layer serving as a selective layer and a support substrate, respectively (Figure 1b,c). This paper will comprehensively review the latest developments, especially within the last five years of these application in water purification and desalination, alongside cutting-edge research advancements [10].

## 2. Electrospinning Technique and Process

### 2.1. Electrospinning Technique

In the late 16th century, William Gilbert began to describe the behavior of magnetic fields and electrostatic phenomena. The process of ES was patented by J.F. Cooley in 1900 and by W.J. Morton in 1902. In 1934, Formalas invented an experimental apparatus for the preparation of polymer fibers by electrostatic force. The experimental device vividly reveals how polymer solutions form a fluid between electrodes. It was first time volume production of fibers in high voltage static electricity was described in detail and is now mostly recognized as the beginning of ES technology in fiber preparation. During the 1930s to 1990s, with significant attention directed towards ES exploration, many researchers applied for a series of patents. However, few succeeded. Nonetheless, given the rapid development of nanotechnology in recent years, ES technology has thrived and prospered and is once again thriving and prospering. This brings about renewed hope for this technology in scientific research, and also renews industrial circles’ hopes for this technology. Throughout this period, the development of ES has undergone four stages. The first stage focused on the spinnability of different polymers, the influence of process parameters on the diameters and properties of fibers during the spinning process, and the optimization of the process parameters. The second stage concentrated on the diversity of the nanofibers and their structure. The third stage specialized in applying electrospun fiber in the fields of energy, environment, biomedicine and optoelectronics, whereas the fourth stage paid heed to the large-scale manufacturing of electrospun fibers.

**Figure 2:**
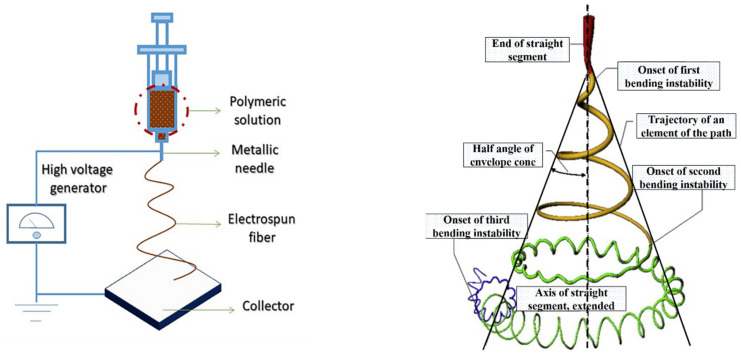
Schematic representation of the ES process. Reprinted with permission from Ref. [27]. 2020, Elsevier.

ES technology has the advantages of simple manufacturing devices, low spinning costs, a wide range of spinnable materials, and precise and controllable processes [23,24]. Because of these advantages, ES technology has become one of the universal methods for the effective preparation of nanofiber materials. The electrospun nanofibers bear several remarkable properties such as a small diameter, large surface area, high aspect ratio, unique physiochemical properties, and flexibility [25]. The ES technique device consists of a high-voltage supply device, a syringe tube with small diameter needles, and a metal collecting plate/roller (Figure 2). The temperature and humidity of the environment should be kept stable. During the ES process, a superior high voltage electrical force is utilized on the polymer-solvent system (polymer solution or polymer melt). After that, the polymer solution is injected from the spinneret by overcoming surface tension of the solution under a superior high electric field force. A polymer jet is principally affected by surface tension and electrostatic force during the stretching process. In this process, the stretching of fibers is affected by various forces such as surface tension, Coulomb repulsion force, electrostatic force viscoelasticity, gravity, and air resistance [26]. Subsequently, collectors collect a strip of fiber in a specific dimension when the jet is stretched, and the solvent evaporates through the air. During the ES process, the bending instability results in the high stretching of the fiber.

### 2.2. Effects of Electrospinning Parameters

Nanofibers with variable dimensions or morphologies can be manufactured under different ES conditions [28,29]. The polymer fiber diameter and morphology are affected by both the polymeric solution properties as well as the process parameters. The main factors influencing the polymer solution properties include polymer weight and architecture, solution concentration, polymer viscosity, solution conductivity, and surface tension and solvent. Operating parameters include electric potential, polymer solution flow rate, the distance between the capillary and collector, needle gauge, collector, ambient temperature, humidity, and air velocity in the chamber. The relationship between surface properties and ES parameters is listed in Table 2.

## 3. ENMs with a Nanofiber Layer as the Selective Layer

ENMs with a controllable fiber diameter and pore size, narrow pore size distribution, high and tunable porosity, low tortuosity, and controllable thickness—as well as a customized structure—have drawn great attention from researchers and the industrial purification field. The parameters of the ES process, such as the concentration of the polymeric solution, solvents ratio, applied electric voltage, the ambient temperature, and so forth, have been proved to necessitate critical optimization. One polymer (or co-blended polymers) with inorganic additives to fabricate ENMs is commonly utilized in the water purification. Modification and variations are further required to provide versatile physicochemical properties and better performance in the water desalination process. Herein, the influencing factors, such as electrospinning conditions, mixed matrix solution, and surface modification, are discussed.

### 3.1. Conventional ENMs

Polyacrylonitrile (PAN) [58], polysulfone (PSF), polyvinylidene fluoride (PVDF) [59], polyurethane (PU) [60], polyvinyl alcohol (PVA) [61], polyethersulfone (PES) [62], fluoropolymer, poly(vinylidene fluoride-co-hexafluoropropylene) (PVDF-co-HFP, PH), polystyrene (PS) [63], and polydimethylsiloxane (PDMS) [64] are favored for their adjustable fiber diameters, impressive membrane porosities and uniform pore sizes [65]. It is a universal preference that these polymers be electrospun into ENMs with the pore size range of 0.1–1.0 μm, which significantly affects the removal of particles exceeding 1.0 μm. These membranes can be regulated under different operating conditions to enhance the performance of ENMs.

#### 3.1.1. Polymer Blending

The selection of polymers will affect the parameters, such as surface tension, electrical conductivity, and viscosity of the ES solution [66]. ES needs to overcome the solution surface tension for spinning, and reducing the surface tension facilitates the formation of fibers without beads. Too low a viscosity may lead to the interruption of polymer filaments and polymer droplets, while too high a viscosity makes it difficult to extrude the polymer. The polymer solution conductivity affects the fiber diameter. The choosing of the appropriate one or mix of the polymer solutions significantly affects the properties of ENMs. 

ENMs with different properties are required for various applications, and ENMs prepared from a single polymer make it difficult to meet the demand. In terms of co-blending polymers, ES is a technique to combine polymers with different properties into one reservoir, electrospun together to improve the performance of the membranes [67]. The nanofibers made of a polymer blend can also give rise to new applications due to the integration of functions originating from individual components. Silk (SF) has excellent mechanical and binding resistance properties. Poly(ethyleneimine) (PEI) is a hyperbranched cationic polymer which was shown earlier to improve the antibacterial activity of resins. Ugur et al. [68] combined SF, PEI, and PMMA with ES technology to successfully prepare antibacterial- and mechanically enhanced nanofiber membranes.

The coexistence of phenol and salt in wastewater increases its toxicity and has a detrimental effect on the ecosystem. Therefore, it is necessary to prepare a membrane that is highly permeable to phenol and impermeable to water and salt to treat phenol-laden saline wastewater. PDMS, as a superhydrophobic material with a low surface energy and surface density, is widely used to improve membrane hydrophobicity. However, simple methods for the preparation of superhydrophobic PDMS membranes are still to be explored. Ren et al. [68,69] used poly(methyl methacrylate) (PMMA) as a carrier polymer for superhydrophobic ENMs to creatively fabricate PDMS/PMMA ENMs to treat phenol-laden saline wastewater. The result showed that an increase in PDMS/PMMA mass ratio led to a rise of solution viscosity, where the cosolvent was composed of equal volumes of DMF and THF. The optimized membrane exhibited a high WCA of ~163°, a good permeation flux of ~39.6 Lm^−2^h^−1^ and an excellent salt rejection of ~99.96%. 

**Figure 3:**
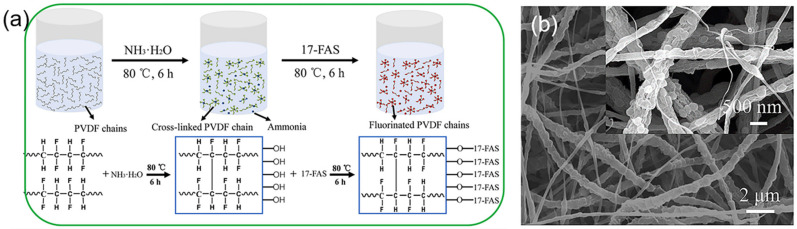
(**a**) Schematic illustration of PVDF chains crosslinking induced by NH_3_·H_2_O and the 17-FAS grafting onto PVDF polymer chains; (**b**) FE-SEM images of PVDF ENMs through a fluorinated, self-roughened process and thermal treatment. Reprinted with permission from Ref. [72]. 2020, Elsevier.

Zhang et al. [70] incorporated polyaniline (PANi) into styrene block copolymer polystyrene-b-(ethylene-co-butene)-b-styrene (SEBS) dissolved solution for SBES/PANi ENMs, which accordingly obtained excellent strain recovery capability and thermal stability compared with the pristine SEBS ENMs. The SEBS/PANi ENMs had a remarkable corrosion resistance to stainless steel, even under large tensile deformation. Zhu et al. [71] newly developed Janus fibrous membranes by incorporating NH_3_·H_2_O, 17-FAS, and PVDF powder into PVA/PAA solutions to fabricate PVA/PAA ENMs (Figure 3a,b). When the simulated hypersaline wastewater composed of 3.5 wt% of NaCl, 0.1 g of SDBS and 1.0 g of lubricating oil was desalinated, a high water flux of over 27 Lm^−2^h^−1^ and a high desalination efficiency of ~100% were achieved after heat treatment.

#### 3.1.2. Other Fabricating Parameters

As illustrated in Table 2, operating conditions consequently worked effectively on nanofibers and ENMs. The polymer solution (such as volume and concentration) and operating parameters (such as temperature and pH) are explored separately to improve the performance of the nanofiber layer. 

**Figure 4:**
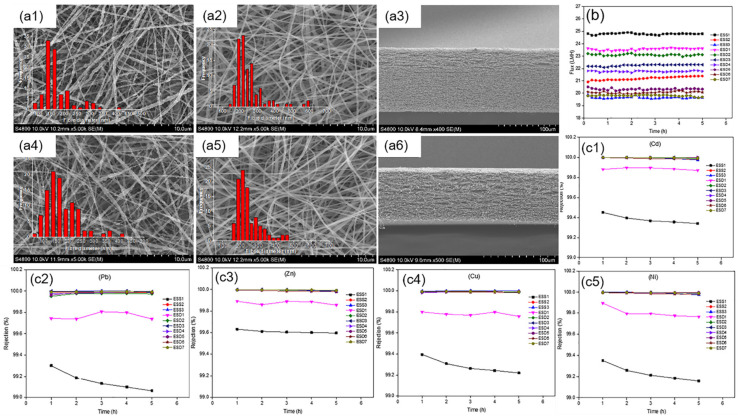
(**a**) SEM images for the top layer (**a1**,**a4**), bottom layer (**a2**,**a5**), and cross-section (**a3**,**a6**) of the prepared PVDF ENMs; (**b**) Permeate flux of PVDF ENMs (ESD1–7); (**c**) Rejection of Cd, Pb, Zn, Cu and Ni (**c1**–**c5**). Reprinted with permission from Ref. [73]. 2018, Elsevier.

Attia et al. [73] fabricated superhydrophobic dual-layer PVDF ENMs with different membrane thicknesses by varying the volume and concentration of the polymer solution (Figure 4). The multiple heavy metal rejection of the prepared ENMs was all above 99%. Moreover, dual-layer PVDF ENMs possessed a higher permeate flux above 23 Lm^−2^h^−1^ for 2500 ppm total heavy metal concentrations and exhibited a mechanical performance and a slight reduction in LEP compared with the single-layer ENMs in the same volume of polymer solution by air-gap membrane distillation. 

Wang et al. [74] first adjusted the membrane porosity using a facile hot-pressing method. The membrane porosity decreases from ~86% to ~34% after the hot-pressing process, increasing the rejection of 0.2 μm particles from 0 to 100%. Compared to conventional microfiltration media, these membranes possessed a high flux value of ~10,320 Lm^−2^h^−1^MPa^−1^ and low degrees of fouling in the long run. Barroso-Solares et al. [75] reported a post-pressing on neat PMMA fibers before utilizing water-in-oil emulsion separation for homogeneous distribution. In addition, flexible pH is also the critical point at which to adjust the surface morphologies of ENMs. Shekarabi et al. [76] studied the pH effect on PAN ENMs with an aluminum precursor added in spinning solution, based on an ionization topic and a metallic chemical structure for fast heavy metal ions adsorption. Electrostatic excretion between Cr^6+^ and membrane surface was raised, with the pH values increasing from 3.5 to 11. Hence, the adsorption was negligible until it reached a pH value of 11, and Cr^6+^ was adsorbed notably and was precipitated at pH 3.5 by taking place on the solid surface. 

### 3.2. Mixed Matrix ENMs

Recently, the fabrication of mixed matrix membranes with dispersive nanoparticles incorporated into the continuous polymer matrix is gaining importance for its advantages compared with polymeric and inorganic membranes. Preparation of the mixed matrix nanofiber selective layer by incorporating inorganic nanomaterials, organic crosslinking agent, and carbon-based nanomaterials into polymer solution to improve the ENMs performance are illustrated below [77].

#### 3.2.1. Inorganic Metal Incorporation

Inorganic metal modification consists of metal nanoparticles (Ag, Fe) and metal oxide materials (Al_2_O_3_, TiO_2_, MOFs) [78]. Morphologies and structures of the composite mixed matrix ENMs are altered, hence varying from that of the original. This generates excellent water purification efficiency in heavy metal ions adsorption, organic dyes removal, antibacterial applications, oil–water separation, and some membrane distillation processes.

Ag nanoparticles of dimensions ranging from several nanometers to tens of nanometers, with their excellent properties of killing and inhibiting bacteria and microorganisms [79], have sparked great interest in mixed matrix electrospun selective layer formation in membrane production for water desalination [80]. However, when directly added to the polymer solution, Ag nanoparticles will agglomerate, and hardly disperse on the electrospun nanofibers. Therefore, the reduction reaction between Ag^+^ and Ag nanoparticles is selected to solve this problem. Yuan et al. [81] added Ag^+^ into the PVA solution and heated it to 150 °C to make the obtained PVA ENMs composite insoluble in water. The Ag^+^ was reduced by PVA, amalgamated together and simultaneously modified on the membrane mat with the ES process. The optimum adsorption performance of the obtained ENMs towards Hg^2+^ in water was ~248 mg/g in 333 K. 

**Figure 5:**
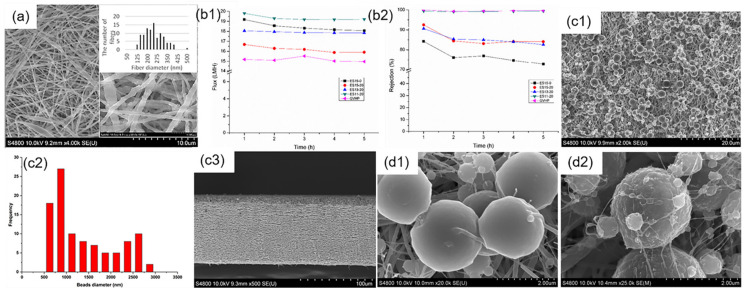
(**a**) SEM images of optimized fibers; (**b1**,**b2**) Flux data and rejection percentage as a function of time for the commercial and the synthesized membranes; Reprinted with permission from Ref. [82]. 2017, Elsevier. (**c1**–**c3**) SEM images of top surface, beads diameter and cross-section of the optimized PVDF/Al_2_O_3_ ENMs; (**d1**,**d2**) SEM images of beads without and with 30 wt% Al_2_O_3_ nanoparticles. Reprinted with permission from Ref. [83]. 2018, Elsevier.

In addition to metal ions, metal compounds such as Al_2_O_3_ and TiO_2_ are widely used for incorporation into polymer solutions. Attia et al. [82] fabricated hydrophobic PVDF/Al_2_O_3_ ENMs through a one-step production. Superhydrophobic Al_2_O_3_ nanoparticles functionalized by branched hydrocarbon were firstly incorporated into polymer solution to facilitate hydrophobicity and surface roughness. Cationic surfactant HTAB was used to reduce the beads of fibers. The WCA was about 150°, higher than the pure PVDF membrane’s temperature of 132° (Figure 5a,b). The synthesized membranes were advantageous compared to commercial membranes, where the heavy metal rejection was up to 99.4%, with a permeate flux of about 20 Lm^−2^h^−^^1^. Their group [83] then combined electrospray with an electrospinning technique to fabricate superhydrophobic PVDF/Al_2_O_3_ ENMs with beaded surface features (Figure 5c,d). The synthesized membrane possessed a superior permeating performance of ~18.6 Lm^−2^h^−1^ and ~99.99% rejection of multiple heavy metal elements, with high liquid entry pressure, water contact angle and a low sliding angle.

TiO_2_ has also attracted much attention as it can improve the hydrophilicity of nanofibers, accelerate the interaction between water and membranes, and even has abilities to inhibit certain bacteria. Razzaz et al. [84] fabricated the chitosan/TiO_2_ ENMs by TiO_2_ incorporated into chitosan dissolved in 3% acetic acid solution. Controlling the concentration of TiO_2_ nanoparticles was essential for the obtainment of uniform and non-defect membranes. As the 2 wt% TiO_2_ nanoparticles were mixed with 7 wt% chitosan, agglomeration and coagulation could not occur in the structure of the composite ENMs, which increased the adsorption sites of the membrane. At a pH level of 6, the maximum adsorption capacities for Cu^2+^ and Pb^2+^ ions of the composite ENMs were ~710.3 and ~579.1 mg/g, respectively. This was due to the electrostatic interaction between the heavy metal ions and nanofibers. Besides, the addition of zeolite to the nanofibers enhanced the adsorption capacities towards heavy metal ions [85].

#### 3.2.2. Inorganic Nonmetal Incorporation

Inorganic nonmetallic nanomaterials such as SiO_2_ [86], carbon nanotubes (CNTs), zeolite [87,88], graphene, and graphene oxide (GO) were incorporated into the polymeric solution for functional improvements.

Silica nanoparticles added to the polymer solution have the ability to improve the mechanical strength of nanofibers, increase the glass transition temperature of ENMs, and prevent the appearance of beads throughout the ES process [89,90]. Hou et al. [91] dissolved the polyvinylidene fluoride-co-hexafluropropylene (PVDF-HFP) in a cosolvent of DMF/acetone, and added SiO_2_ emulsion into the reagent to obtain PVDF-HFP/SiO_2_ ENMs. With the mass ratio of SiO_2_ increasing, though the membrane porosity decreased gradually, the pores shrunk while the thickness of membranes was enhanced, resulting in the improvement of salt rejection efficiency. The WCA was over 150°, owing to the rougher hydrophobic surface. The highest permeate flux was up to 48.6 kg/m^2^h and the rejection of NaCl was maintained at nearly 100% after 240 h of continuous operation of the membrane distillation process.

CNT membranes are expected to be used as high-performance self-assembly membranes in water purification, and their molecular sieving performance mainly depends on the effective pore size of the membranes [92]. CNTs in polymer composites can enhance the tensile modulus and strength of polymers, which are immobilized in nanofibers by ES to enhance the mechanical toughness of the composite nanofibers [93]. Excellent mechanical strength and modulus are attributed to the strong electrostatic attraction between electropositive CNT nanotubes and negative polymer matrix. The robust composite nanofiber membrane is promising for water purification processes. CNTs and its modified versions are considered ideal nanofillers because of their high thermal and mechanical stability and their light weight. Yan et al. [94] prepared a hierarchical carbon nanofiber membrane by electrospinning PAN/TPA solution, followed by pre-oxidation and carbonization, to achieve high-performance desalination under sunlight, even in acid/alkali conditions. The ENMs performed well in interfacial solar desalination of 1.36 kgm^−2^h^−1^ evaporation rate and >99.9% rejection of main ions in seawater with markedly improved surface roughness and surface wettability.

The idea of using industrial waste materials (that might pollute the environment) to make composite membranes for water treatment may bring a new impetus to the field. Fly ash, as a relatively abundant and inexpensive adsorbent, has attracted a lot of attention in the field of water treatment. It can be used to adsorb different toxic substances, such as arsenic, dyes, volatile organic compounds, and many more [95,96]. Pant et al. [97] prepared a stable silver-doped fly ash/polyurea (Ag-FA/PU) nanocomposite multifunctional film using fly ash particles (FAPs) through a simple one-step ES process. Colloidal solution of PU with FAPs and Ag metal precursor was used to fabricate nanocomposite spider-web-like membrane using an ES process. The films can be used for adsorptive removal of dyes and arsenic, destructive removal of microorganisms, and removal of particulate impurities by single membrane filtration using a continuous filtration process.

**Figure 6:**
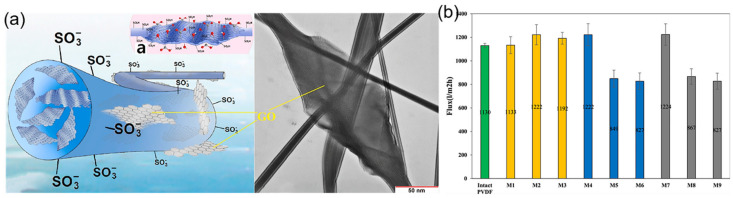
(**a**) Schematic diagrams and TEM images of S-PVDF/PVDF/GO nanofibers; (**b**) Water flux of PVDF ENMs with CNTs in different concentrations. Reprinted with permission from Ref. [101]. 2017, Elsevier.

Graphene and GO possess most of the properties of carbon nanotubes. Their exceptional chemical and thermal stability and rich hydrophilic functional groups contribute to their being attractive nanofillers [98,99]. Woo et al. [100] incorporated graphene into a PH solution and fabricated the superhydrophobic PH/graphene ENMs. As 5 wt% graphene was added, the porosity reached ~88% and WCA was up to ~162°. Graphene in the nanofibers increased the roughness of the membrane surface, thereby affecting high surface hydrophobicity, and thermal and chemical stability additionally in the 60 h membrane distillation operation. The composite ENMs showed a high water flux at ~22.9 Lm^−2^h^−^^1^ and maintained a high salt rejection of ~100%. Ahmadi et al. [101] prepared a novel electrospun nanofiber membrane by ES using sulfonated polyvinylidene fluoride (S-PVDF)/PVDF and S-PVDF/PVDF/graphene oxide (GO) with negative charge as raw materials (Figure 6). WCA of the optimized ENMs was as low as 77.1° and high purity water flux was ~1222 Lm^−2^h^−1^. The irreversible fouling was controlled by enhanced hydrophilicity under 41% and the water flux recovery ratio was about 59%.

The shape and porosity of nanofiber membranes are crucial in water filtration. The use of spider web morphology nanofibers or nanoweb fibers in the field of water filtration has garnered considerable attention in recent years. Pant et al. [97] prepared spider-web-like silver-doped fly ash/polyurea (Ag-FA/PU) nanocomposite membranes by an ES process. The spider-web-like nano-meeting structure enhances the adsorption of carcinogenic arsenic (As) and toxic organic dyes, as well as improves antimicrobial performance to reduce the bio-fouling of membrane filters. Inspired by a natural spider-web-like structure, Xing et al. [102] prepared a new type of atrazine molecularly imprinted nanofibrous membrane (A-MNM). The spider-web-like structure provided sufficient surface area for the formation of imprint recognition sites. Interestingly, during the separation, the target molecules are captured by the A-MNMs, just like a spider web entangles its prey in nature. The spider-web-like morphology nanofibers or nano-net fibers further broadens the application of ENMs in the field of water purification.

#### 3.2.3. Organic Incorporation

In contrast to various inorganic additives, there are fewer applications of organic additives due to various rigorous conditionalities, which, in general, are covalent organic frameworks (COFs), metal organic frameworks (MOFs), poly-cyclodextrin [103,104], β-cyclodextrin, and their modifiers [105]. 

MOFs are a class of nanoporous materials composed of central metal clusters or ions and organic ligands, which possess a large specific surface area, a high porosity, a low thermal conductivity, and ordered nanopores [106]. In recent years, MOFs have attracted significant interest for their role as nanofillers in pressure-driven liquid separations to drastically improve separation performances by providing additional water channels [107]. In 2016, Zuo and Chung first introduced MOFs into membranes for membrane distillation, which led to a breakthrough in new applications for ENMs [108]. Shooto et al. [109] discovered that the diameter of PVA nanofibers decreased when the content of MOFs increased. This subsequently contributed to the increase of solution conductivity by MOFs. The prepared PVA/MOFs ENMs exhibited a higher uptake capacity of Pb^2+^ in aqueous solution over commonly used activated carbon. Efome et al. [110] dispersed pre-hydractivated Zr-based MOF-808 (Zr-MOF-808) in PAN solution for PAN/Zr-MOF-808 ENMs. Zr-based MOF-808 was synthesized and hydractivated according to Li et al. [111] in 2015 with the mechanism of electrostatic interaction and metal ions binding to adsorption sites on MOFs. The obtained ENMs showed optimized adsorption capacities of ~225.1 mg/g and ~287.1 mg/g for Cd^2+^ and Zn^2+^ respectively. In the same year, they [112] added Fe-based MOF-808 into PAN solutions similarly to form PAN/Fe-MOF-808 ENMs. The synthesized ENMs can dispose~395 mL of the 100 ppb Pb^2+^ solution, while maintaining adsorption capacity of above 90% for Pb^2+^ after 4 cycles. 

COFs are a class of robust two- or three-dimensional extended network materials known for their artistic structures and for their potential for a wide range of applications [113,114]. Wang et al. [115] combined a new type of COF-SCU1 (firstly reported by Sichuan University [116]) with PAN powder to form a DMF solvent for electrospinning, thereby obtaining PAN/COF-SCU1 ENMs. The composite PAN/COF-SCU1 ENMs retained the strong adsorption of COFs and prevented the breakdown from pure COFs as adsorbents. The optimized adsorption of tetracycline antibiotics was above 84–99% in a neutral aqueous environment, which was far more than the 10% of pure PAN ENMs, and declined lower than 30% after five continuous cycles.

### 3.3. Surface Modification

The internal structure of the membrane, created by the random accumulation of nanofibers as well as surface modification, can affect both pore size and liquid entry pressure. A surge of composite ENMs with a modified nanofiber layer via organic grafting of additional functional groups has attracted great interest to achieve a higher separation performance.

Wang et al. [117] grafted hyperbranched polyethylenimine (HPEI) and glycidol onto PAA ENMs to form PAA/HPEI-glycidol ENMs via the reaction between the carboxylic group on PAA with the amine groups in HPEI, followed by the ring-opening of amine terminal groups with glycidol. The formation of the complexation compound formed boron and hydroxyl groups in glycidol, which vastly improved boron adsorption. The maximum boron adsorption capacity of the optimized ENMs achieved up to 5.7 mmol/g and remained approximately 94% after 10 operation cycles. Zhao et al. [118] demonstrated a facile preparation of branched polyethylenimine (bPEI) grafted PAN ENMs with a refluxing approach facilitating the reaction between cyano groups of PAN and bPEI. It has been proven that the modified PAN/bPEI ENMs gained notable improvements in the adsorption of Cr^6+^ by achieving up to 637.5 mg/g, making it superior to many other adsorbents. The resulting membrane could cause the concentration of Cr^6+^ in water to drop to the WHO standard, below 0.05 mg/g, indicating its significant use in removing toxic metal ions. 

**Figure 7:**
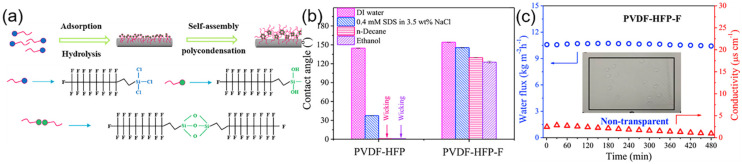
(**a**) Schematic illustration of vapor deposition fluorination; (**b**) Contact angles of DI water and other liquids on PVDF-HFP and PVDF-HFP-F membranes; (**c**) Conductivity and water flux verses time of the PVDF-HFP-F membrane in DCMD. Reprinted with permission from Ref. [120]. 2020, Elsevier.

Distinct methods of pre-treatment, taking the vapor activation method as an example, have been newly developed. It has been demonstrated that the surface properties were labile when exposed to organic solvents or high humidity [119]. Wu et al. [120] fluorinated PVDF/PH ENMs without surface activation via vapor deposition to lower the surface energy of the membrane (Figure 7). It was understood that, as compared with traditional dip-coating, vapor deposition is more convenient in large-scale fabrication. The optimal fluorinated ENMs showed robust anti-wetting properties during membrane distillation in the trial. Moreover, Liu et al. [121] used the solvent vapor to partially melt CA/PVDF ENMs and promoted contact points among the nanofibers for physical crosslinking, which was testified to be simple yet effective to enhance the mechanical strength of the resulting membranes. 

## 4. ENMs with Nanofiber Layer as Supporting Substrates

The nanofiber layer can also be used as the supporting substrate of ENMs, owing to its unique advantages of a good surface-to-volume ratio and splendid porosity conducive to the selective layer’s growth [122]. A selective layer was generally formed on the nanofiber layer by secondary-electrospinning, inorganic deposition, polymer coating, and interfacial polymerization, which are crucial in determining the path of fluid flow and separation efficiency [123]. Moreover, structure matching, such as thickness and porosities of the supporting layer and the selective layer, should be precisely tailored by independent adjustment to achieve the best performance for the intended application.

### 4.1. With the Selective Layer via Secondary-Electrospinning

Dual-layer hydrophobic composite ENMs with two kinds of nanofiber layers were superior candidates in the water desalination process, especially membrane distillation, owing to its unique advantages of well-connected inner pores and low mass transfer resistance. It was reported that dual-layer ENMs, generally composed of hydrophobic/hydrophilic or superhydrophobic/less hydrophobic layers, exhibit better performance in membrane distillation than single-layer hydrophobic ENMs of the same thickness [124,125]. Besides, using a hydrophobic/hydrophilic dual-layer hollow fiber membrane for long-term DCMD operation demonstrated fewer pore wetting issues [126,127].

**Figure 8:**
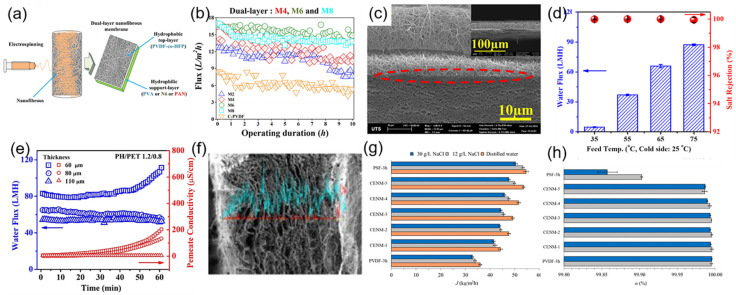
(**a**) Schematic diagram of the fabrication process of dual-layer ENMs; (**b**) permeance performance of the prepared ENMs; Reprinted with permission from Ref. [126]. 2017, Elsevier. (**c**) cross section SEM images of the optimized dual-layer ENMs; (**d**,**e**) permeating performance and stability test in different conditions of thermal and strain; Reprinted with permission from Ref. [128]. 2020, Elsevier. (**f**) cross-section SEM images and EDX analysis of the optimized dual-layer ENMs (CENM-5); (**g**,**h**) permeating performance of dual-layer ENMs. Reprinted with permission from Ref. [129]. 2017, Elsevier.

Woo et al. [126] prepared dual-layered ENMs composed of hydrophobic polyvinylidene fluoride-co-hexafluoropropylene (PH) top nanofiber layer with different supporting hydrophilic nanofiber layers for distillation desalination (Figure 8a–c). The heat-pressed PH/nylon-6 exhibited high hydrophobicity and enhanced mechanical and thermal properties. Large porosity (~85.3%) promoted a high flux of ~15.5 Lm^−2^h^−1^ and salt rejection above 99%. An et al. [128] assembled the PH nanofiber layer and PET microfiber layer via convectional electrospinning to fabricate hierarchical composite PET/PH ENMs (Figure 8b–e). During membrane distillation, the highly hydrophobic PH nanofibers presented an enhanced anti-wetting property as well as a high salt rejection. Concurrently, PET microfibers provided an improved mass transfer and heat insulation. The relatively high permeability (~65.9 Lm^−2^h^−1^) and high salt rejection (>99.99%) were optimized for the composition and thickness of membranes. Khayet et al. [129] assembled the hydrophobic bottom layer PVDF and hydrophilic top layer PSF nanofibers via electrospinning at different times successively to prepare dual-layered ENMs (Figure 8f–h). The prepared PVDF/PSF ENMs exhibited an excellent desalination performance with a flux of ~53.60 Lm^−2^h^−1^, and an NaCl rejection of ~99.99%, which is visibly higher than the traditional ENMs reported in direct contact membrane distillation. Moreover, Hou et al. [130] fabricated biomimetic PTFE/PVA ENMs via sol-gel and electrospinning methods for anti-oil-fouling membrane distillation. Their asymmetric wettability attributed by the hydrophobic PTFE nanofiber layer as the bottom layer and the highly hydrophilic PVA-Si-GA layer as the top layer, helped to sustain a stable operation over 50 h with 1000 mg/L crude oil as foulant.

### 4.2. With the Selective Layer via Inorganic Deposition

ENMs obtained with the selective layer via inorganic deposition from the nanofiber layer can usually enhance membranes’ thermodynamic and chemical stability. Properties of such hierarchical membranes are strongly affected by the selectivity of nanomaterials, which are commonly applied to heavy metals, some toxic ions adsorption and oil–water separation [131].

**Figure 9:**
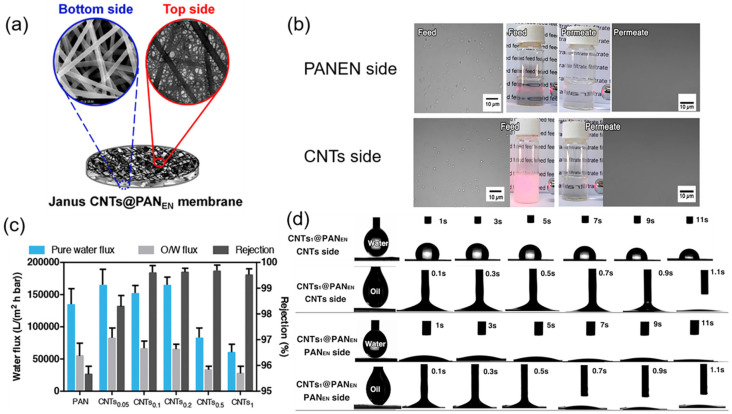
(**a**) Schematic illustration and SEM images of Janus PAN/CNTs ENMs; (**b**) separation results by two sides of Janus PAN/CNTs_0_._5_ ENMs; (**c**) pure water flux, O/W flux and water rejection by the pure PAN and PAN/CNTs ENMs; (**d**) spreading behaviors of water and oil droplet on two sides of PAN/CNTs_0_._5_ ENMs. Reprinted with permission from Ref. [133]. 2017, Elsevier.

Yan et al. [132] cohered CNT layers onto the PVDF ENMs by the heat-pressing process. The CNTs were dispersed in ethanol to spray without agglomeration. The prepared superhydrophobic PVDF/CNT ENMs exhibited the highest water flux of ~28.4 Lm^−2^h^−1^, with above 26 h of steady performance in a vacuum membrane distillation. Jiang et al. [133] deposited hydrophobic single-side CNTs on the hydrophilic PAN nanofiber layer through a vacuum filtration process in order to fabricate Janus PAN/CNTs ENMs. Various concentrations of the PAN and CNT solution generated different properties of hydrophobicity/hydrophilicity in both sides of the membrane (Figure 9). The optimized oil rejection was increased to exceed 99.5% followed by the incorporation of CNTs with a high flux of ~120,000 Lm^−2^h^−1^MPa^−1^.

Electrospray technique, an electrostatic atomization process, is utilized for the beaded fiber structure without compromising pore architecture or structural integrity [134]. Guo et al. [135] fabricated superhydrophobic PVDF-co-HFP ENMs with monolayer nanofiber and nanospheres by one-step biaxial electrospinning and an electrospray method. Nanospheres were dispersed uniformly on each nanofiber by electrospraying. Huang et al. [136] used the electrospraying technique on chitosan-rectorite nanospheres from the aminated PAN(APAN) nanofiber layer via shoulder-to-shoulder electrospinning. The aminating modification was carried on the surface of PAN by DETA. Chitosan was extruded into nanospheres by electrospray and was assembled on APAN nanofibers to enlarge the inner three-dimensional space and to facilitate the water flux with the resulting undermined flow resistance. With visible improvements seen among the amine groups in APAN regarding the adsorption process, the APAN-CA ENMs exhibited at least a double increment during the adsorption process towards Pb^2+^ in contrast to the original PAN-CS membranes. 

### 4.3. With the Selective Layer via Polymer Coating

Various materials, such as chitosan [137], PES [138], DETA, PVA [139], and PDMS [140], with or without nanoparticles such as TiO_2_ incorporated, were coated as selective layers on the surface of nanofiber layers, forming new types of ENMs to be applied in ultrafiltration and oil–water separation processes specifically [141]. 

Zhao et al. [142] coated amino-rich hydrothermal DETA onto the PAN nanofiber layer after an efficient hydrothermal carbonization method. Amino groups of DETA availably reacted with the carboxyl groups of the carbonaceous PAN nanofiber layer to form an amide bond. The adsorption capacity of polluting Cr^6+^ and 2,4-dichlorophenoxyacetic acid were ~290.7 mg/g and ~164.5 mg/g, respectively, which were superior to the pure PAN ENMs. Ren et al. [143] coated TiO_2_ precursor sol on the surface of the electrospun PVDF basis for improvements in hydrophobicity and pore structure of the membrane surface. The TiO_2_ precursor sol was obtained from ethanol, 2,4-pentanedione, perchloric acid, and titanium (IV) isopropoxide mixtures, subsequently modified by fluoride. The resultant properties of high hydrophobicity (~157.1°), considerable wetting resistance of under 0.15 MPa, a well-distributed pore size of 0.8 μm, a reasonable surface porosity of ~57%, and a modest membrane thickness of 55 μm made the modified PVDF ENMs a competitive alternative option for DCMD. Besides, Vanangamudi et al. [144] conducted a study by coating 18 wt% PVDF onto the hydrophilic nylon-6,6/chitosan nanofiber layer to form the Janus membrane. High hydrophilicity was created due to the intermolecular hydrogen bonding interaction between the membrane surface and water molecule, reaching the highest water permeance ~5420 Lm^−2^h^−1^MPa^−1^. Levels above 93% BSA were rejected for permeation through the membrane with decreased pore sizes at ~3930 Lm^−2^h^−1^MPa^−1^ liquid flux in the filtration process.

### 4.4. With the Selective Layer via Interfacial Polymerization

Besides deposition and coating, selective layers prepared via interfacial polymerization (IP) can also be formed on the surface of nanofiber layers to fabricate ENMs, which are mainly used for osmosis and nanofiltration processes. The typical material of this selective layer is polyamide (PA), which is prepared through the reaction of polyamines and acyl chloride [145]. 

**Figure 10:**
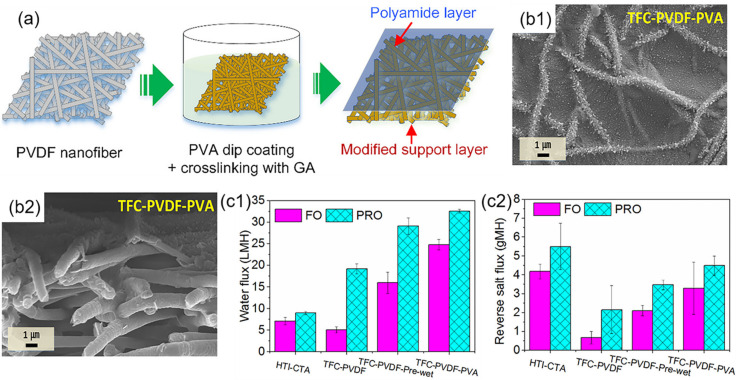
(**a**) Schematic diagram of TFN C PVDF/PVA ENMs; (**b**) FESEM images of PVDF/PVA nanofiber layer and TFNC PVDF/PVA ENMs for top surface (**b1**,**b2**); (**c**) FO performance of commercial HTI-CTA (**c1**) and prepared TFNC PVDF/PVA ENMs (**c2**). Reprinted with permission from Ref. [148]. 2017, Elsevier.

In the forward osmosis process, Tian et al. [146] post-covered PA layer from M-phenylenediamine (MPD) and 1,3,5-trimesoylchloride (TMC) on the PET/PVA nanofiber layer that is formed from electrospinning. The diffusive resistance was primarily decreased due to the low tortuosity and inner interconnected pores in the nanofibrous support layer. These open pore structures and high roughness facilitated the water molecules to move across the membrane. The optimal water flux of 0.5 M NaCl solution was ~30.6 Lm^−2^h^−1^ in the forward osmosis process. Shokrollahzadeh et al. [147] fabricated a PA rejection layer on the top surface of the PSF/PAN nanofiber layer based on the IP reaction between MPD and TMC monomers. Due to the high porosity of about 84.3% and a large number of interconnected pores in the membrane, the structural parameters were optimized to facilitate the formation of the uniform PA layer, decrease the internal concentration polarization (ICP) effect, and reverse the salt flux through the desalination. High porosity promoted the mass transfer across the membrane to allow the water flux to reach ~38 Lm^−2^h^−1^ towards the NaCl solution in the forward osmosis process, which was superior to the utilization of the traditional substrate, fabricated by phase inversion. Park et al. [148] deposited the PA layer from MPD and TMC on the hydrophilic pretreated PVDF/PVA nanofiber layer (Figure 10), with the nanofibers being relatively rough and full of nano-sized pores. The PVDF/PVA nanofiber layer not only enhanced the hydrophilicity towards the liquid, but also reduced the ICP effect and reverse salt flux in the osmosis process. With 0.5 M NaCl as the draw solution and DI water as the feed solution, the water flux could reach up to 34.2 Lm^−2^h^−1^ and the reversed salt flux was as low as 0.13 g/L. 

Besides, in the nanofiltration process, Mahdavi et al. [149] studied the PET nanofiber layer as substrate, p-phenylenediamine (PPD), and triethylamine (TEA) in the aqueous phase, and TMC in the oil phase to fabricate PA film as the selective layer used in the nanofiltration process. The trifluoroacetic acid (TFA) and dichloromethane (DCM) were used as solvents to sculpt morphologies of PET by dissolution. With the higher ratio value of the concentration of TFA/DCM, a better electrical conductivity of the polymeric solution could be achieved, and smaller diameters of nanofibers could be obtained. This induced the unique and flat morphology of the nanofibers, as well as numerous interconnected structures throughout the mat. Thereafter, the PA selective layer was synthesized onto the surface of the PET layer. The water flux could reach ~34 Lm^−2^h^−1^ in 0.5 MPa, which is four times higher than other conventional nanofiltration membranes, while the rejection of Na_2_SO_4_ was about 93%. In addition, Kaur et al. [150] studied two interfacial polymerization methods onto the PVDF nanofiber layer. These two methods subsequently led to different surface topologies and different osmotic selectivity. In the first method, PVDF nanofiber layers were immersed in the aqueous and organic phases in succession. In method B, however, the order was reversed. Compared with the first method, ENMs prepared by the second method had a more uniform PA layer with a rejection rate of ~80.7% and a flux of ~0.5 Lm^−2^h^−1^ for 2000 ppm MgSO_4_ under 0.5 MPa. 

## 5. Future Scope

Although advancements in academic research and certain applications have been reported in the field of ENMs for water treatment, there are still challenges to be confronted.

As to the electrospinning process, appropriate conditions for electrospinning and modifications are required for better manufactures and performance. Nanomaterials that are cheap, non-toxic and environmentally friendly are being focused upon. Moreover, the preparation approaches of ENMs are generally reported to be time-consuming and to have a low yield on the lab-scale. Therefore, it is necessary to build more efficient equipment for the mass production of high-quality ENMs.

As to ENM fabrication, mechanical stability, wettability and nanoscale selectivity are imperative parameters for efficient performance in water treatment. The internal bonding force in nanofibers and covalent bonding between nano-layers have considerable attention paid to them, especially in dual-layer composite ENMs, which consequently hampers the structural stability. Once the exfoliation occurs, ENMs were out of action immediately and thus lost both permeability and selectivity. Mixing matrix electrospinning solutions was proven to be a practical strategy to improve ENMs properties, and more explorations of new dopants are required for better performance as well; however, there are still problems of uniform distribution of particles in the polymer structure (agglomeration phenomenon of nanoparticles), cavity formation, immiscibility of blending polymers, and weak interactions between particles with the polymer matrix and the construction of mixed matrix membranes. Specifically, the physical and chemical stability of mixed-matrix membranes in water, including nanoparticles’ detachment from the membrane surface and the incompatibility between nanoparticles and the organic polymer matrix, should be taken into account when developing new nanomaterials. 

As to the operation and filtration of the membrane, durable stability, mechanical stability, and operational sustainability in realistic conditions should be crucially evaluated in the long term. The majority of reported ENMs were tested in a laboratory-simulated water environment in the short term, however, which differed from the complex water environment in realistic application. Efficient separation to complex systems with different physical and chemical properties remains to be desired and must urgently be explored; it cannot be simply resolved by ENMs yet. In addition, there is still a lack of industrial production equipment. Though certain applications were carried out in some reports, most of the promising applications are still limited to the laboratory or to the primary stage of development. More attention should be paid to the transformation of theoretical innovation into practical production. 

Furthermore, the interaction between actual contaminants and ENMs should be clearly understood so that various membrane technologies can be applied extensively. It is necessary to further study the manufacture of ENMs with thin, robust, and highly selective active layers and recycling stability on the actual industrial scale.

## 6. Conclusions

Decent and reliable technology for water purification is urgently needed throughout the world. ENMs, with their unique features and well-organized architecture, display an up-and-coming prospect of future adsorption and filtration systems to efficiently exclude pollutants and macro/small molecules in water purification.

In this review, the current status and application aspects of traditional ES techniques are well-illustrated, with special attention on the effects of operating parameters on nanofibers for reference. ENMs, with the nanofiber layers serving as selective layers, are mainly produced in three ways: conventional ENMs, mixed matrix ENMs, and surface modification via grafting. For the production of mixed matrix electrospinning solutions, inorganic nanomaterials and organic materials are both furnished by some examples which play different roles in bacterial inhibition, dye removal, separation of oil/water systems, and so on. Such hierarchical membranes’ efficiency is apparently improved by various modifiers, nanoparticles, cross-linked organic reagents, and active biopolymers. However, nanofibers’ direct work as a selective layer has a relatively limited scope of utilization in low-pressure driven process like ultrafiltration, and microfiltration, as well as in thermal-driven process like membrane distillation, due to its poor mechanical strength. Therefore, typical nanofiber layers like PAN, PVDF, and PSF nanofibers and so on are widely installed as a supporting substrate, fixed with an additional skin layer by inorganic deposition, polymer coating, and interfacial polymerization to meet the demand of water treatment in different conditions. All of these contribute to the separation performances of organic pollutants, heavy metals, and antifouling performance in water treatment. It is considered that the ENM market has enormous potential for further development and is anticipated to boom in the immediate future for water purification.

## Figures and Tables

**Table 1:** Comparison of different fabrication techniques of fibers.

Techniques	Technical Process	Materials	Benefits	Drawbacks	Ref.
Electrospinning	Polymer solution or melt flow and transform into polymer jet from spinneret under a high voltage electric field (usually 6 kV above); When the jet is extended and solvent is vaporized, the nanofibers in specific dimensions are collected by collectors	Polyester, polyamide, polyvinyl alcohol, polyacrylonitrile, polyurethane, polyp-benzoyl, p-phenylenediamine etc.	High-productive; Simple for operation; Products with advantages of exceedingly porous, well interconnected, huge internal surface area, three-dimensional fibrous system and extraordinarily separation ability	Vulnerable mechanical strength	[11,12]
Dry spinning	Polymer solution is extruded from spinneret and cooled down by hot air stream; Polymer trickles turn to fibers in solid state during solvent extraction	Cellulose acetate, polyolefin, polyvinyl chloride, vinylidene chloride spandex etc.	Wide range of raw materials; Pollution-free and no solvent recovery problems	Low yield	[13,14]
Wet spinning	Polymer solution is extruded from spinneret and then solidified into fibers through coagulating bath; Fibers are extruded and collected	Polyacrylonitrile, polyvinyl alcohol, polyvinyl chloride, viscose, polypyrrole, conductive polyaniline, inorganic nanofibers like carbon nanotubes etc.	Uniform structure and high quality of fibers; Directional fibers can be collected; Possible to produce inorganic nanofibers for separation application	Low speed in spinning; Short Fibers	[15,16]
Emulsion spinning	Polymer powder is dispersed in easy-spinning carrier solution; Carriers dissolve in high-temperature processing; Polymer powder is melted or sintered into successive fibers	Polytetrafluoroethylene, ceramic, silicon carbide, monox, chloroethylene etc.	Specificality in the polymers unsuitable for dry spinning and wet spinning	Presence of structural defects and impurities; Low structural stability;	[17,18]
Melt spinning	Polymer particles are heated, melt and extruded into spinneret; Polymer jet is impinged and cooled down by hot air stream to produce fine fibers; The fine fibers are collected in a collector screen	Polyolefin, polyamide, polyester, polyvinyl chloride etc.	Simplicity and efficiency; No solvent recovery problems	Requirements of high voltage electric field and temperature; None nano-sized fiber	[19,20]
Phase separation spinning	Polymer solution of two or multiple components is exposed to the gelation temperature to get the gel; Phase separation of solvent and polymer occur due to temperature and pressure changes; Nanofibers are generated and collected after solvent extraction and matrix drying	Polyacrylonitrile, poly (2, 6-dimethyl p-phenyl ether), polypropylene, polyvinyl alcohol	Feasibility on the polymers difficult for electrospinning and melt blowing; Mass production;	Limited range of polymer-solvent system; Difficult solvent recovery; Low structural stability; Rare polymers with good gelation ability	[21,22]

**Table 2:** Effects of different conditions on the nanofibers in electrospinning technique.

Sorts	Factors	Influences	Ref.
Polymer solution	Concentration	Viscosity and surface tension of polymer solution increase as the concentration increases; Diameters and pore sizes of nanofibers increase as the solution concentration increases; Instable nanofibers with a low linear density are produced by polymer solution at a low concentration	[30,31]
	Polymer molecular structure	Smoother surfaces and a larger diameter are caused by a branched polymer compared with the linear polymer; Bead-free structures are manufactured by linear polymers	[32,33]
	Polymer molecular weight	Smoothness of nanofibers is increased as the polymer molecular weight increases	[34,35]
	Viscosity	Diameters and pore sizes of nanofibers increase as the polymer viscosity increases; The production of nanofibers is directly influenced	[36,37]
	Conductivity	Diameters decrease and pore sizes increase as the conductivity increases; Bead-formed fibers are caused by a higher conductivity	[38,39]
	Surface tension	Diameters decrease and pore sizes increase as the surface tension increases; Impurities are hard to avoid when surfactants are utilized to reduce the tension	[40,41]
	Solvent volatility	Wet fiber, molten fiber, or even negligible fiber collection are the result of solvent volatility; Highly volatile solutions may result in intermittent spinning due to polymer solidification at the tip of the spinneret; A proper solvent can make the produced fiber uniform and stable	[42,43]
Fabrication conditions	Electric potential	Larger and faster tensile of the solution droplets as the voltage increases; Droplet splitting ability is enhanced, leading to a larger diameter of nanofibers	[44,45]
	Flow rate of polymer solution	Diameters and the beads of nanofibers increase with the higher flow rate; Pore sizes vary due to the different flow rate	[46,47]
	Distance between spinneret and collector	Diameters of nanofibers reduce with the larger distance; Beads appear and the products turn unstable on account of the larger distance	[48,49]
	Syringe Needle gauge	Diameters increase and pore sizes decrease as the nozzle diameter increases; Productivity is enhanced by the smaller diameter of the needle	[50,51]
	Collector	Flat, cylindrical, and prismatic collectors are popular	
Ambient conditions	Temperature	Solvent volatilization and nanofiber solidification are accelerated by the increase in temperature; Electrical stretching of the fluid jet is early terminated by temperature increases; The instability of nanofibers is enhanced due to the decrease in solution viscosity and surface tension by temperature increases	[52,53]
	Humidity	Thinner, less sticky and bead-formed structures are caused by the higher humidity, as well as larger diameters and pore sizes	[54,55]
	Air velocity in the chamber	Nanofibers are enabled to be collected on the collector layer by layer when increasing air flow rate, consistent with the spinning direction; Instability in the ES process is caused by the increased velocity of cross flow air	[56,57]

## Data Availability

Not applicable.
